# Update on the safety of second generation antipsychotics in youths: a call for collaboration among paediatricians and child psychiatrists

**DOI:** 10.1186/s13052-016-0259-2

**Published:** 2016-05-21

**Authors:** Simone Pisano, Gennaro Catone, Stefania Veltri, Valentina Lanzara, Marco Pozzi, Emilio Clementi, Raffaella Iuliano, Maria Pia Riccio, Sonia Radice, Massimo Molteni, Annalisa Capuano, Antonella Gritti, Giangennaro Coppola, Annarita Milone, Carmela Bravaccio, Gabriele Masi

**Affiliations:** Department of Mental and Physical Health and Preventive Medicine, Child and Adolescent Neuropsychiatry Division, Second University of Naples, 80131 Naples, Italy; Child Neurology and Psychiatry Unit, Center for Rare Diseases, Department of Pediatrics, Catholic University, Rome, Italy; Scientific Institute IRCCS Eugenio Medea, 23842 Bosisio Parini, Lecco, Italy; Unit of Clinical Pharmacology, CNR Institute of Neuroscience, Department of Biomedical and Clinical Sciences, L. Sacco University Hospital, Università di Milano, 20157 Milan, Italy; Department of Pediatrics, Hospital “F. Veneziale”, Isernia, Italy; Unit of Clinical Pharmacology, Department of Biomedical and Clinical Sciences L Sacco, L. Sacco University Hospital, Università di Milano, 20157 Milan, Italy; Faculty of Education Science, University Suor Orsola Benincasa of Naples, Naples, Italy; Campania Regional Centre for Pharmacovigilance and Pharmacoepidemiology, Department of Experimental Medicine, Second University of Naples, 80131, Naples, Italy; Child Neuropsychiatry, Faculty of Education, Suor Orsola Benincasa University, Naples, Italy; Clinic of Child and Adolescent Neuropsychiatry, Department of Medicine and Surgery, S. Giovanni di Dio and Ruggi d’Aragona Hospital, University of Salerno, Fisciano, Italy; IRCCS Stella Maris, Scientific Institute of Child Neurology and Psychiatry, Calambrone Pisa, Italy; Department of Translational Medical Sciences, University Federico II of Naples, Via Pansini 5, 80131 Naples, Italy

**Keywords:** Children, Adolescents, Antipsychotics, Adverse events

## Abstract

During the past decade, a substantial increase in the use of second generation antipsychotics (SGAs) has occurred for a number of juvenile psychiatric disorders, often as off-label prescriptions. Although they were thought to be safer than older, first generation antipsychotics, mainly due to a lower risk of neurological adverse reactions, recent studies have raised significant concerns regarding their safety regarding metabolic, endocrinological and cardiovascular side effects. Aim of this paper is to update with a narrative review, the latest findings on safety of SGAs in youths. Results suggest that different SGAs may present different safety profiles. Metabolic adverse events are the most frequent and troublesome, with increasing evidences of heightened risk for type II diabetes mellitus. Results are discussed with specific emphasis on possible strategies of an active monitoring, which could enable both paediatricians and child psychiatrists to a possible prevention, early detection, and a timely management of such effects.

## Background

During the past decade, a substantial increase has occurred in the use of second generation antipsychotics (SGAs) for a number of psychiatric disorders in paediatric patients [1, 2]; the phenomenon is worldwide, although its magnitude varies a lot among countries. For example, in Europe and particularly in Italy the increase is much lower than in the United States (U.S.) [3, 4]. SGAs are prescribed both as in- or off-label medications for various conditions, such as schizophrenia spectrum disorders, irritability and aggression in autism spectrum disorder or intellectual disability, tics or Tourette’s Disorder, mood disorders (mainly bipolar), conduct disorders and eating disorders [2, 5–9]. Polypharmacy with more than one SGA and with other psychotropic drugs is also common (about one third, in U.S. samples) [4, 5]. Clozapine was the first SGA to be released, and thereafter Risperidone, Olanzapine, Quetiapine, Aripiprazole, Ziprasidone, Paliperidone and Asenapine have followed. Second generation antipsychotics block D2 dopamine receptors, similarly to first generation antipsychotics (FGAs), but with lower receptor occupancy. In addition, they block serotonin receptors in the cortico-limbic pathways. For this reason, they were thought to be safer than FGAs (e.g. Haloperidol), mainly due to a better neurological profile [10, 11], although recent studies have raised significant concerns regarding their adverse events related to the metabolic syndrome (weight gain/obesity, hypercholesterolemia, hypertriglyceridemia, hyperlipidemia, hyperglycemia/hyperinsulinism, hypertension) [12–14]. These adverse effects are more frequent in youths than in adults, probably due to less prior SGAs exposure [12, 15]. Other adverse effects were reported, such as hyperprolactinemia, cardiovascular-related events (e.g. cardiac arrhythmias, prolonged QT interval, and orthostatic hypotension), and neurological disturbances (acute and tardive dystonia, neuroleptic malignant syndrome, Creatine Kinase elevation) [16]. In 2011, recommendations for paediatric SGAs use were published by both the AACAP (American Academy of Child and Adolescent Psychiatry 2011) and the Canadian Alliance for Monitoring Effectiveness and Safety of Antipsychotics in Children (CAMESA) [17, 18]. Each recommendation focuses on a combination of different baseline and periodic monitoring of: 1) patient history and physical examination; 2) measurement of height, body weight, or body mass index (BMI); 3) blood pressure; 4) fasting plasma glucose and metabolic variables; and 5) potential, precautionary inclusion of an electrocardiogram (ECG) to identify QTc prolongation [[17, 18]; see also [19] for a summary]. Despite the growing awareness and evidence-based data regarding SGAs safety issues in youth, national monitoring programs are still lacking, and mental health professionals do not seem to adhere correctly to current guidelines [19–21]. Adverse events from therapeutically-intentioned use of antipsychotics still cause thousands of child and adolescents’ emergency department visits annually in the U.S., with significantly higher risk compared to other psychotropic medications, such as stimulants or antidepressants [22]. Despite these concerns, inconsistent monitoring patterns are reported for children assuming SGAs. Rodday et al. [19] found that two thirds of clinicians report a careful assessment of patient history, height and weight, blood pressure, fasting plasma lipids and glucose, but only 23 % report a regular monitoring of waist circumference, and only 12 % monitor cardiac functioning with an electrocardiogram. This is consistent with a study by Connolly et al., according to which insufficient rates of metabolic screening tests are performed, mainly blood glucose level [21].

Aim of this review is to update the evidence on the safety of SGAs in paediatric patients, with specific focus on those that can be prevented, managed or at least blunted with a careful monitoring. This review analyses relevant papers (mainly large prospective and retrospective cohort studies) published from January 2011 to January 2016, after the release of the two guidelines from AACAP and CAMESA. In the text, we will highlight the nature of the study (RCT = randomized controlled trial, ES = epidemiological studies, OS = observational studies), to improve readability. It is important to underline that RCTs may not be powered/appropriate to detect rare or late-onset adverse effects, as compared to large cohort and/or long term observational studies. Results are reported in a qualitative manner, rather than systematically.

### SGAs and weight gain

Since the issuing of guidelines on monitoring and management of antipsychotic-related weight gain [17, 18], more recent studies [23–26] confirmed previous findings [9–12, 27, 28] regarding the different risk with different SGAs. The risk seems higher for Olanzapine and Clozapine, medium for Risperidone, and lower for Quetiapine, Aripiprazole and Ziprasidone [12, 25, 29, 30]. A Dutch study assessed cross-sectionally and, for a subset of patients, longitudinally, children receiving SGAs (82 % Risperidone), for a mean 6-month follow-up (OS) [31]. Compared to a control sample of untreated children, patients receiving SGAs presented significant overweight and obesity (OR = 3.1 and 3.6, respectively), and significant SGA-dependent increase in weight-z (+0.77 vs. -0.1) and BMI-z (+0.8 vs. +0.05) measures. Another study, with a 4-month follow-up, showed a greater weight increase with Risperidone, as compared to FGAs (+3.7 kg vs. + 0.9 Kg), and +13 kg and +4 BMI-z, with respect to untreated subjects, irrespective of drug exposure time (OS) [32]. A relevant study of 4280 adults and 179 adolescents, followed for up to 6 months, investigated the effect of age on Olanzapine-dependent weight gain [33]. The risk was markedly higher in the adolescents, with an average of +11.2 vs. +4.8 Kg and 89 vs. 55 % patients with clinically relevant weight increases, as compared to adults (OS). Short-term effects were investigated in several randomized controlled trials. In a sample of bipolar adolescents receiving Risperidone, body weight increase was two-fold that with Valproic Acid, while BMI increased 3.5-fold after 1 month of treatment (RCT) [34]. Olanzapine treatment for 1.5 months increased weight by 4.2 Kg, compared to placebo, with 31 % patients presenting a relevant weight gain (>7 %) (RCT) [35]. In patients with irritability in the context of Autism Spectrum Disorder receiving Aripiprazole for 2 months, weight increase significantly (0.9 mean Kg in those with prior exposure to antipsychotics, 1.2 mean Kg in naïve ones) and relative risk (the ratio of the probability of an event occurring in an exposed group divided by the probability of the event occurring in a comparison, non-exposed group) of a clinically relevant weight gain was 4.6 as compared to placebo users, with drug-naïve patients (82 %) presenting the highest risk (post hoc analysis of two RCTs) [36]. Regarding Quetiapine, a first study (RCT) [37] reported a detrimental effect after 1 month of therapy, in terms of weight gain (+1.8 to +2.2 Kg), number of patients with relevant weight gain (+11 to +16 %), and increased BMI z-score (+0.2 to +0.3). In the continuation study (4-month follow-up) (OS), 18 % of the study population experienced significant weight gain [38]. A relevant study including only drug-naive patients with mixed diagnoses, treated with Risperidone, Olanzapine and Quetiapine, and followed-up for 6 months, showed that all treatments were associated with an increase of all body measures (weight, BMI, waist circumference) (OS) [23]. More specifically, children of normal weight decreased from 84.5 to 64.4 % (prevalently with Risperidone and Olanzapine), while obese children increased from 5 to 8.6 %. In a comparison among the three SGAs, Olanzapine led to the greatest weight gain, compared to Risperidone (at 3 and 6 months) and Quetiapine (at 6 months), with dose-dependency found for Risperidone and Quetiapine. Dose-dependency was not found in another 7-months study on schizophrenic children and adolescents, investigating Risperidone, Quetiapine and Olanzapine (OS) [39]. No differences among SGAs emerged, with an overall BMI increase of 1.5, and 38 % patients with clinically relevant weight gain. An 18-months study on Risperidone evaluated the effects of maintenance vs. discontinuation vs. SGA switch **(**OS) [40]. Compared to patients who continued Risperidone, neither those who discontinued, nor those who switched to other SGAs (a small subsample of very different drugs) decreased their BMI, body fat mass, waist circumference, and prevalence of obesity [40]. A 24-months observation of psychotic adolescents, treated predominantly with Risperidone, Olanzapine and Quetiapine, demonstrated a greater increase in BMI and BMI z-score among those receiving Olanzapine (+4.3 and 1.3 vs. +2.5 and 0.7 with Quetiapine, and. +1.8 and +0.5 with Risperidone) at 6 months, while no differences among SGAs were found at 24 months (OS) [24]. A study with similar duration, comparing Risperidone and Aripiprazole in children and adolescents with Autism Spectrum Disorder, found similar significant increases for both drugs (+2.4 BMI and +0.5 BMI-z per year with Risperidone, and +2.1 BMI and +0.6 BMI-z per year with Aripiprazole) (OS) [41]. Another study on a sample of patients with first psychotic episode (mean age 27 years) compared Olanzapine, Risperidone and the FGA Haloperidol (OS) [42]. At the study end-point of 36 months, patients gained an average of 12 Kg, and 78 % patients presented a clinically relevant weight gain, with 31 % having a BMI >30. Weight rapidly increased during the first year of treatment (85 % of the total weight gain), then prevalently stabilized over time. An association was found between the use of Olanzapine and earlier weight gain. Interestingly, at 36 months weight gain was associated with a positive psychiatric course [42].

### SGAs and diabetes

The rapid weight gain and metabolic abnormalities determined by SGAs have been associated with the increased risk of Diabetes Mellitus type 2 (DM2) (for a review see Galling et al. [43]). Moreover, Teff et al. demonstrated that antipsychotics are associated with a post-prandial insulin dysregulation, independently from weight gain [44]. Such data were confirmed through large epidemiological studies [45, 46]. A retrospective cohort study of the Tennessee Medicaid program compared 28,858 youths who started antipsychotics (87 % SGAs, 40 % Risperidone) and 14,429 controls, who started other drugs (mood stabilizers, stimulants, antidepressants, α-stimulants and benzodiazepines) (ES) [47]. Data showed that the first group had a 3-fold increased risk to develop DM2; the risk increased significantly with the increase of cumulative dose, and remained high after 1 year from discontinuation. Results were similar when the cohort was limited to children 6 to 17 years of age [47]. Another study by Shon et al. reported similar results, with a more than 2-fold increased risk to develop DM2 in antipsychotic users; interestingly, the risk difference between those receiving and not receiving SGAs was evident after 4 to 6 months of treatment (ES) [48]. More recently, a U.S. study focused on the incidence of DM2 in drug-naïve SGA users (ES) [49]. The sample included 1,328,985 youths aged 10 to 18 years (107,551 assuming SGAs, 38.9 % Risperidone). The incidence of DM2 was 0.4 and 0.2 % respectively in SGA initiators (I) and non-initiators (NI) (I: prevalence 38/10,000, NI: prevalence 25/10,000). Authors concluded that SGAs increased the risk of DM2 (O.R. 1.51, C.I. 1.35–1.69, *p*< 0.001). The mean time between the first SGA exposure and DM2 diagnosis was 13.5 months (SD 9.2). Furthermore, the concomitant use of stimulants did not reduce the risk, whereas the concurrent use of an antidepressant further increased it. Authors showed that, compared with Risperidone, newer antipsychotics were not associated with a decreased risk [49]. In the study by Arango et al., a significant increase in the number of patients experiencing hyperglycaemia was found in the Risperidone group, compared to other SGAs (OS) [23]. A recent meta-analysis confirmed that the cumulative risk and the incidence rate ratio were significantly greater in antipsychotic-exposed youth, compared to healthy and psychiatric controls, with Olanzapine presenting the greater cumulative risk [50]. From another perspective, Galler et al. explored glycaemic and metabolic control in youths with type 1 diabetes with and without antipsychotics. They found a higher HbA1c level among SGAs users (ES) [51].

### SGAs and dyslipidemia

SGA use can lead to dyslipidemia [25, 29, 52]. Triglycerides and cholesterol increase occurs early, and may even precede weight gain, showing an independent molecular effect, in addition to the weight related-effect [25]. A study including adolescent and adult patients with firsts psychotic episode receiving Olanzapine, Risperidone and the FGA Haloperidol (OS) [42] found weight, cholesterol and trygliceride levels rapidly increased during the first year of treatment (85 % of the total weight gain), then prevalently stabilized over time. In the study by Arango et al. (OS) [23], Risperidone was associated with an increase in triglycerides, and Olanzapine with an increase in total cholesterol and low-density lipoproteins. In this study, Quetiapine showed no impact on triglycerides and cholesterol, but other studies reported an increase of such parameters [30, 39]. Aripiprazole and Ziprasidone seem to cause dyslipidaemia less than other SGAs, but data are far from being conclusive [53].

### SGAs and prolactin

The dopamine blockade on the anterior lobe of the pituitary gland due to SGAs is often associated with hyperprolactinemia. Raises in prolactin (PRL) levels may cause gynecomastia, galactorrhea, irregular menses, and amenorrhea in women, sexual dysfunction (decreased sexual desire, erectile-ejaculatory dysfunction, orgasmic dysfunction, vaginal dryness), and reduced fertility [54]. The induced hypogonadotropic hypogonadism, together with low oestrogen and testosterone levels, may lower bone mineral density, and cause osteoporosis [55]. Children and adolescence are at higher risk for hyperprolactinemia, probably due to an age-related decrease in dopamine receptors [14, 54, 55]. Risperidone and, to a lesser extent, Olanzapine and Ziprasidone, appear to have the largest propensity for prolactin elevation, while Quetiapine and Clozapine seem to be neutral as regards PRL levels [26, 30, 55, 56], and Aripiprazole often lower PRL levels, due to its partial dopamine agonism [57]. Regarding Risperidone, a meta-analysis by Pringsheim et al. [29] found significant increases in PRL levels from baseline to endpoint in Risperidone-treated compared to placebo-treated children, with a mean difference of 44.57 ng/ml (*p*< 0.00001). Another study revealed hyperprolactinemia during Risperidone treatment in up to 65.8 % of patients, usually without signs or symptoms [57]. The rise in PRL is dose-dependent, and usually occurs in the first 4–8 weeks of treatment [55]. In a recent comparative trial between Risperidone and Quetiapine, the latter showed a less pronounced effect on prolactin levels (RCT) [58]. Less is known on the long-term consequences of hyperprolactinemia. Based on data of two studies with 1-year follow-up [59, 60], hyperprolactinemia seems not to affect growth and sexual maturation, but to increase the risk for gynecomastia (at least regarding Risperidone) [61]. Although it cannot be totally ruled out, the causal relationship between prolactin-raising antipsychotics and prolactinomas is still missing [25]. Regarding Aripiprazole, it resulted to induce a marked reduction of prolactin levels. In a study by Safer et al., 60 % of those treated with Aripiprazole showed suboptimal prolactin levels as compared to 8 % of unmedicated subjects. The prolactin decrease was found to be more prominent with higher doses and longer treatment durations (OS) [62]. The effect of prolonged low prolactin levels on brain and physical development is mostly unknown and warrant future studies.

### SGAs and cardiovascular safety

Electrocardiogram (ECG) abnormalities and arrhythmias -Torsades des pointes (TdP) have been reported in patients on FGAs and SGAs [63, 64]. An absolute QTc interval of >450 ms or an increase of 60 ms from baseline are often used as threshold requiring clinical attention [65, 66], but arrhythmias are most often associated with values of 500 ms or more [53]. Intervals of 440 to 460 ms in men and 440 to 470 ms in women are considered borderline [67]. However, evidences to support a causal pathway from antipsychotics use to QTc prolongation and risk of TdP and sudden death are less conclusive [53, 68]. QTc prolongation is partly dose-related, although a genetic disposition may contribute to antipsychotic-induced arrhythmogenesis, as up to 10 % of individuals developing TdP presents mutations associated with the congenital long-QT syndrome [68]. Signals for QTc prolongation have been detected in a large database study for Risperidone and Ziprasidone (ES) [64]. Correll et al. (OS) [69] measured the frequency of baseline prolongation of QTc in 811 psychiatric paediatric inpatients. QTc duration was 440 ms or greater (range 441–481 ms) in 16 patients (1.97 %). Compared with patients with normal QTc, similar rates of utilization of antipsychotics (43.8 vs. 40.8 %) and daily chlorpromazine equivalent doses (165 ± 110 vs. 168 ± 218 mg) were found in patients with prolonged QTc, while a correlation was found between obesity and QTc prolongation. Germanò et al. (OS) [70] assessed the arrhythmogenic risk of Risperidone and Aripiprazole in paediatric patients. Despite a small increase in mean QTc in Risperidone group, no clinically meaningful effects were found for both drugs. Ho and colleagues found no significant cardiac effect of Aripiprazole in patients with autism spectrum disorder treated for irritability for up to 14 weeks (OS) [71]. Gulisano et al. assessed the cardiovascular safety of Aripiprazole compared to the FGA Pimozide. At the end of the 24 months observation period, they found no effect on cardiac conduction for Aripiprazole, whereas Pimozide significantly increased the QTc interval (OS) [72]. More recently, Jansen et al. [66] performed a meta-analysis on QTc changes during antipsychotic treatment, including randomized and open label clinical trials. This meta-analysis included 5,423 patients with QTc data (mean age 12.8 ± 3.6 years, 32.1 % of female). Within group, from baseline to endpoint, Aripiprazole significantly decreased the QTc interval by 1.44 ms, whereas a modest increase was found with Risperidone (+1,69 ms, CI = +0,67 to +2,60, *p* = 0.001) and especially with Ziprasidone (+8,74 ms, CI = +5,19 to +12,3, *p* = 0.001). However, compared to pooled placebo, none of the investigated antipsychotics caused a significant increase of the incidence of QTc prolongation. These findings are substantially consistent with findings from adults [73]. A recent study including 216 paediatric patients on Olanzapine, Quetiapine and Risperidone, followed-up for 12 months, failed to report increases in QTc or in heart rate; moreover, there were no QTc values >500 ms at any of the time assessments (OS) [74]. Ziprasidone was found to be associated with a significant prolongation of QTc, but not QTc dispersion, in a dose- and level-independent way, in a quarter of youth in a longitudinal study of 29 patients (OS) [75]. Based on these data, the risk of pathological QTc prolongation seems low during treatment in healthy youth. Nevertheless, because individual risk factors interact with medication-related QTc effects, a careful assessment of both medication and patient factors need to be considered when choosing the antipsychotic treatment.

Another possible cardiac adverse event during antipsychotic treatment is myocarditis. It has been described mainly for Clozapine in adults with a frequency up to 3 %; data on youths are lacking, but awareness and caution are required, namely in long-term treatments [76].

### SGAs and neuromotor adverse effects

Movement disorders, namely extrapyramidal symptoms (EPS) were historically the most common antipsychotic adverse effects leading to an emergency department visit. SGAs have been considered at lower risk of EPS, as compared to FGAs. However, the risk is not absent, and children and adolescents seem more sensitive than adults to EPS [11, 16, 29]. These effects include chronic or acute dystonia (sustained involuntary muscle contractions causing twisting and repetitive movements or abnormal postures), parkinsonism (rigidity, tremor, bradykinesia and postural instability), akathisia (a sense of inner unease, manifested by an inability to sit still and a need to move around), and tardive dyskinesia (chronic and persistent abnormal movements such as grimacing, tongue movements, lip smacking, pursing of the lips, excessive eye blinking, rapid and involuntary movements of the limbs, torso, and fingers) [68]. A systematic review and meta-analysis of randomized controlled trials demonstrated a higher risk of EPS for Risperidone, Aripiprazole and Olanzapine, as compared to placebo, whereas Quetiapine and Clozapine seem to be more neutral in this regard [29].

Recently, Carbon et a l. reported the incidence rate of EPS in a large cohort of paediatric patients followed for up to 3 months (OS) [77]. A relatively low incidence of drug-related Parkinsonism (15.2 %) was found, with the highest rate for Aripiprazole (27.2 %), and lowest for Quetiapine (1.5 %). The overall 3-month incidence rate of treatment emergent dyskinesia was 8.28 %, ranging from 4.41 % for Risperidone to 26.6 % for Ziprasidone. The overall 3-month incidence rate of akathisia was 4.8 % without differences among SGAs. In multivariate analyses, a significant effect of drug type was detected for Quetiapine and Olanzapine (low EPS liability) and Ziprasidone (high EPS liability). Drug-induced Parkinsonism was associated with higher doses, older age, and lower baseline functioning, while titration rate was debated as a possible triggering factor. Also polypharmacy has been associated with increased rate of tremor and dyskinesia [77]. Neurological adverse events of Risperidone, Quetiapine and Olanzapine were explored in a sample of 256 subjects naturalistically treated over a time span of 1 year (OS) [78]. Risperidone presented higher scores in dyskinesia and Parkinsonism, while Quetiapine was associated with less neurological adverse events. Younger age, psychotic symptoms and higher cumulative exposure time all increased the risk for tardive dyskinesia.

### SGAs and neuroleptic malignant syndrome

A rare, severe adverse reaction associated with antipsychotic treatment is neuroleptic malignant syndrome (NMS), characterized by muscular rigidity, hyperthermia, autonomic dysfunction, alterations of mental status, myonecrosis, leukocytosis and elevated blood CPK [79]. As recently reviewed, SGAs may cause NMS as well as FGAs, but with lower incidence, lower clinical severity, and more rare fatal outcomes [79]. Findings are similar in children and adolescents. In a case series of 23 NMS cases, the time of onset ranged from immediately to 56 days (mean 8.7 ± 16.2 days), and subjects were predominantly males, and no one of the cases resulted in death or permanent sequelae [80]. Signal detection for NMS was found for Aripiprazole in a database study (ES) [64]. Masi et al. reported on a particular condition that may occur during SGA treatment, named MACKE (Massive Asymptomatic Creatine Kinase Elevation). MACKE is an isolated asymptomatic elevation of CK, not explained by other neurological or medical conditions or environmental events, without other signs of NMS. They suggest that the “real” MACKE undergoes spontaneous remission, and does not represent a prodrome of NMS or rhabdomyolysis. However, in case of a random finding of CK elevation, close monitoring of the patient is warranted [81].

## Discussion

This review substantially supports the validity of existing guidelines (see Tables 1 and 2) [17, 18]. However, it is important to highlight some relevant new evidences. The main finding of this study is that metabolic adverse effects and heightened risk for type II diabetes mellitus are the most frequent and troublesome. Usually, a weight gain of >7 % from the baseline weight is considered clinically relevant [14, 29]. Although the weight gain propensity of SGAs is renown, its extent is striking. Olanzapine and Clozapine seem to promote the highest weight gain, followed by Risperidone, and then Aripiprazole, Quetiapine and Ziprasidone [14, 23, 25]. As different time patterns of weight gain between SGAs have been demonstrated, clinicians should carefully consider the timing of monitoring [23]. Weight markedly increases during the first 3 months of treatment with all drugs, and thus, whatever drug is used, the monitoring should be frequent and rigorous in this phase. Subsequently (3–6 months of treatment), the magnitude of weight gain may tend to decline as compared to the first 3 months with Olanzapine and Risperidone, whereas no further increase should occur with Quetiapine [23]. However, long term data from adults do not indicate typical antipsychotics as safer [82]. Prevalence of a full metabolic syndrome in children and adolescents on SGAs is low [12, 14], but given the paucity of long-term data, we cannot rule out a cascade of events leading from weight gain to full blown of metabolic syndrome. Unfortunately, data on diabetes seem to support this notion [47–50]. The three-fold increased risk for diabetes in SGA users is troublesome, and preliminary data on dyslipidaemia are not reassuring as well. Consistent with guidelines and some recent reviews [43, 83], we strongly advise a baseline and routine glycaemic and lipid profiling to timely detect patients at risk for type II diabetes or metabolic syndrome. This is even more important in case of a family history of diabetes, dyslipidaemia and obesity, or in case of weight gain. Overall, prevention and treatment of metabolic symptoms during SGAs exposure are still unmet needs, especially in youths. Psychoeducational and physical programs have been developed, but data come from adult populations, and these findings still lack translation from research to the clinical practice, or from adult to young populations (cost effectiveness, adherence, etc. should be verified) [84]. Switching to an antipsychotic with lower propensity for weight gain seems to be a good strategy to blunt the metabolic effects, but no randomized trials have yet been conducted, and no SGA is absolutely free from metabolic consequences. Metformin seems to be a promising agent to reduce glucose intolerance, and even weight gain, but data on paediatric populations are still scant [85]. A placebo-controlled trial in children on SGAs reported Metformin to be safe and effective in abrogating weight gain; it decreased insulin sensitivity and abnormal glucose metabolism [86]. Few placebo-controlled studies have explored the positive effect of add-on melatonin in mitigating metabolic side-effects of antipsychotic drugs [87, 88]. Regarding adolescents aged 11 to 18 years, Mostafavi et al. compared 24 bipolar youths receiving olanzapine, lithium carbonate and melatonin, and 24 receiving olanzapine, lithium carbonate and placebo, and melatonin significantly inhibited the rise in total cholesterol levels compared to placebo, while fasting blood sugar and triglyceride showed greater increase in the placebo group, but the differences were not statistically significant [89]. Finally, a single case report suggested that Omega-3 fatty acid supplementation may limit triglycerides and cholesterol increases [52].

**Table 1:** Summary of adverse events sorted by drugs

Adverse events	Clozapine	Olanzapine	Risperidone	Quetiapine	Ziprasidone	Aripiprazole
Weight gain	++	++	+	+/−	+/−	+
Diabetes	++	++	+	+/−	+/−	+
Dyslipidemia	+	++	+	+/−	+/−	+/−
Extrapyramidal symptoms	−	+	++	+/−	++	+
Cardiac effect (mainly Qtc prologation)	+ ^a^	+/−	+	+/−	++	+/−
Prolactin	−	+/−	+	−	+/−	− ^b^

Legend: ++ highest propensity to cause the index adverse event. + propensity to cause the index adverse event. +/− slight propensity to cause the index adverse event or inconsistent findings among papers. − neutral regarding the index adverse event. ^a^risk for myocarditis. ^b^possible lowering effect

**Table 2:** Suggested monitoring pattern and AEs management strategies^a^

Time	What to do	Results	Possible strategies
I Step	General and neurological examination (weight, waist circumference, blood pressure…)	Normal	Plan a careful and tailored monitoring program/psychoeducation on drug side effects/healthy lifestyle, including diet and, when possible, exercise
Baseline			
		Abnormal	
	Careful anamnestic interview about personal and familiar history of: Dyslipidemia, DM2, Obesity, Thyroid dysfunction, Arrythmogenic risk (sudden death, syncope, Prolonged QTc, Brugada syndrome)	Negative History	Regular monitoring
		Positive History	Plan a careful and close monitoring program/psychoeducation on drug side effects/healthy lifestyle/ECG (see below)
	Blood examination for haemachrome, liver function, glucose, insulin and lipid profile, (thyroid function and PRL if possible)	Normal	Regular monitoring/healthy lifestyle
		Glucose, transaminases, insulin and/or lipids significantly increased	Careful and close monitoring/psychoeducation/chose a SGA with lower metabolic impact
	ECG (in case of Ziprasidone or positive personal or familiar history)	Normal ECG and QTc<450 msec	Monitoring
		QTc>450 msec or other arrythmogenic signs	Discuss with paediatric cardiologist about the cardiac safety and the risk- benefit ratio in starting a SGA/if possible chose another drug class or a SGA with lower impact on QTc
II Step	Weight and waist circumference monitoring	Weight gain <7 % of baseline weight	Regular monitoring/healthy lifestyle
1 month control			
		Weight gain >7 % of baseline weight	Careful and close monitoring/Healthy lifestyle/Psychoeducation/if possible switch to another SGA
	Blood examination for haemachrome, liver function, glucose, insulin and lipid profile	Normal	Regular monitoring/healthy lifestyle
		Glucose, transaminases, insulin and/or lipids significantly increased	Careful and close monitoring/Healthy lifestyle interventions/Psychoeducation/if possible switch to another SGA
	PRL related symptoms (galactorrhea, increased breasts volume, sexual dysfunction…) monitoring	- symptoms	Regular monitoring
		+ symptoms	Blood prolactin determination/if possible lower the SGA dose/if possible switch to another SGA/if possible add Aripiprazole/discuss with paediatric endocrinologist the possible add on of a prolactin-lowering drug (cabergoline or bromocriptine)
	EPS and other neurological symptoms monitoring	- EPS	Regular monitoring
		+ EPS	If possible lower dose/if possible add on anticholinergics or benzodiazepines
		NMS symptoms	Hospitalization
	ECG (in case of Ziprasidone or positive personal of family history)	Normal ECG and QTc<450 msec	Regular monitoring
		QTc>450 msec or increase from 60 msec from baseline or other arrythmogenic signs	Discuss with paediatric cardiologist about the cardiac safety and the risk- benefit ratio in continuing a SGA/possible SGA discontinuation
III Step	Weight and waist circumference monitoring	Weight gain <7 % of baseline weight	Regular monitoring/healthy lifestyle
3-, 6- month and periodic (every 6 months)			
		Weight gain still increasing	Careful and close monitoring/Healthy lifestyle/Psychoeducation/if possible switch to another SGA
	Blood examination for haemachrome, liver function, glucose, insulin and lipid profile	Normal	Regular monitoring/healthy lifestyle
		Glucose, transaminases, insulin and/or lipids significant increased	Careful and close monitoring/Healthy lifestyle interventions/Psychoeducation/if possible switch to another SGA
	PRL blood determination	Normal PRL	Regular monitoring
		PRL↑- associated symptoms	Careful and close monitoring/if possible lower SGA dose/if possible switch to another SGA/if possible add Aripiprazole/discuss with pediatric endocrinolgist to add on cabergoline or bromocriptine
		PRL↑ + associated symptoms	
	EPS and other neurological symptoms monitoring	- EPS	Regular monitoring
		+ EPS	If possible lower dose/if possible add on anticholinergic or benzodiazepines
		NMS symptoms	Hospitalization
	ECG (if possible and feasible; mandatory in case of Ziprasidone or positive personal of family history)	Normal ECG and QTc<450 msec	Regular monitoring
		QTc>450 msec or increase from 60 msec from baseline or other arrythmogenic signs	Discuss with paediatric cardiologist about the cardiac safety and the risk benefit ratio of SGA continuation/possible SGA discontinuation

Legend: *AE* adverse effects, *DM2* Diabetes Mellitus type 2, *ECG* electrocardiogram, *NMS* neuroleptic malignant syndrome, *PRL* prolactin, *EPS* extrapyramidal symptoms

^**a**^The table summarizes findings from the present review (and is inspired by previously published guidelines [17, 18]); it is limited to the AEs reviewed in the present review; it would represent a guide for clinicians without replacing their clinical judgement

Regarding EPS, Carbon et al. explored possible differences between SGAs (lowest risk for Olanzapine and Quetiapine, highest risk for Ziprasidone), and warned about polypharmacy and improper high doses [77]. Consistently, Garcia-Amador reported lowest risk for Quetiapine, and highest for Risperidone. Whereas Carbon et al. deemed neuromotor events as not being a major clinical problem, due to their relatively low prevalence and low severity, Garcia-Amador et al. warranted caution (and active monitoring), and pointed out the false belief about the neuromotor safety of SGAs.

Recent data provided new insights on QTc prolongation and cardiac safety [66–73]. Based on empirical evidences, SGAs are relatively safe regarding cardiac concerns, as QTc prolongation is a rare event [69, 74], and its magnitude is often clinically irrelevant [66]. However, although baseline ECG is not required by both guidelines (except for Ziprasidone) [17, 18], we suggest that performing an ECG, if possible and feasible, may be advisable after 3 months of treatment and every 6 months (mainly in case of higher doses, fastest titration, concurrent medications or obesity). Furthermore, a careful anamnestic interview, exploring possible individual factors (personal or familial history of syncope, tachy/brady-cardia, palpitations etc.), and/or familial cases of sudden death, prolonged QTc, Brugada syndrome, and other arrhythmogenic conditions, is warranted. In these conditions, irrespective of the SGA used, baseline and follow-up ECGs are strictly advised after 1 month of treatment and every 3 months (mainly in case of higher doses, fastest titration, concurrent medications or obesity). When QTc prolongation is greater than 450 ms, resting heart rate is >130 beats per minute, the PR interval is >200 ms, the QRS is >120 ms, discussing with a paediatric cardiologist the possible discontinuation of the SGA should be seriously considered.

Guidelines pointed out that routinely prolactin determinations can be avoided in patients without symptoms (gynecomastia, galactorrhea, and amenorrhea) [17, 18]. However, in our opinion, given the uncertainties about the effect of a prolonged, albeit asymptomatic, hyperprolactinemia during development, a baseline PRL, a monthly clinical monitoring of PRL-related symptoms, and periodic PRL assessments (twice per year) are warranted. When hyperprolactinemia is persistently high, especially in youngest patients, a switch to SGAs with lower risk of hyperprolactinemia (Aripiprazole, Ziprasidone, Quetiapine, Olanzapine) should be considered. In alternative, especially when PRL- related symptoms are evident, the associations with dopaminergic medications usually administered for treating hyperprolactinemia (bromocriptine, cabergoline), or with low-dose Aripiprazole, should be considered [90, 91].

## Conclusion

Findings from this review highlight the need for a constant, rigorous and careful monitoring of young patients receiving SGAs. There is need for long-term studies, aimed to improve monitoring by all professionals involved in paediatric (physical and mental) health. Only from studies including large, consecutive, unselected young patients with different gender and age ranges, from different sites, may new information stem to prevent, diminish, or at least timely detect and deal with SGA-related adverse events. It was recently shown that the long-term effectiveness of SGAs in youths needs to be improved, with high rate of clinicians-decided and patients-decided discontinuation [92]. A possible improvement strategy is to increase our knowledge on therapeutic drug monitoring, but to date empirical information on paediatric populations is scant [93, 94]. Close paediatrician-child psychiatrist collaboration should be the first action to achieve this improvement.

## Abbreviation

AACAP: American Academy of Child and Adolescent Psychiatry 2011
CAMESA: Canadian Alliance for Monitoring Effectiveness and Safety of Antipsychotics in Children
DM2: diabetes mellitus type 2
ECG: electrocardiogram
EPS: extrapyramidal symptoms
FGA: first generation antipsychotics
MACKE: massive asymptomatic creatine kinase elevation
NMS: neuroleptic malignant syndrome
PRL: prolactin
SGA: second generation antipsychotics
